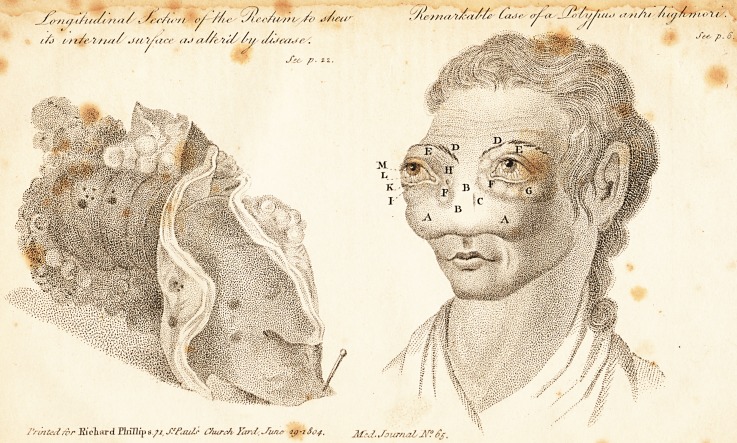# Observations on the Severe Dysentery, *as It Existed on Board the Lord Duncan East Indiaman during a Voyage to Bengal, in 1802—4. In a Letter to* John Hunter, M.D. F.R.S. *Physician to the Hon. East India Company*

**Published:** 1804-07-01

**Authors:** James Atkinson


### OBSERVATIONS ON THE SEVERE DYSENTERY, *as it existed on board the Lord Duncan East Indiaman during a Voyage to Bengal, in* 1802—4. *In a Letter to* JOHN HUNTER, M.D. F.R.S. *Physician to the Hon. East India Company. By* JAMES ATKINSON.

( Continued from Vol. xi. pp. 508—518. )

CASE II. Mr. W——, passenger, of a rigid fibre, had been much exposed to the sun in an open boat, and afterwards to heavy showers of rain. On the 18th of October, when I first saw him, he had laboured under a low fever three or four days, but with no distinct remissions. He is of a melancholy disposition, and is subject to fits of insanity. He had taken an emetic the first thing, then the bark, which with difficulty was retained, though assisted by opium. For two days the stomach was very irritable. I found him with brown furred tongue and lips, cold skin, and great despondency. His pulse was weak and about 120. Prescribed draughts of camphor, tinct. opii gtt. xv. and tinct. cinchonæ ʒiij. to be taken every four hours, and wine ℔ij. per diem.

19th. The pulse more full and regular, with a soft skin. He is continually changing posture, and is very low spirited.

20th. The tongue not so black, but still foul. Feels no particular pain. Very obstinate, and often refuses his medicine. Pulse regular. I now find that he has had frequent stools, scanty and bloody, since yesterday, with tenesmus. He does not appear feverish in the smallest degree, but has a cold and dry skin. Former medicine discontinued. ℞. Ant. tartariz. gr. j. calomel. gr. iij. ext. opii, gr. ß. ft. pil. to be taken every three hours.

21st. Tongue still foul. Took the pills regularly, from which he found great relief. Skin soft and moist. In the night he called for his medicine, and slept soundly at intervals. He has not passed any blood since yesterday evening, but his stools are still slimy, and attended with tenesmus. Complains that his gums are a little sore.*

22d. Dysentery easier. Tongue whitish, but perfectly clean round the edges. In good spirits; gentle perspira-

* Whenever the gums become slightly affected, the disease almost invariably abates, and the calomel is immediately withdrawn. Medicine and food to invigorate the system are still continued.

tion. Omit the pills during the night, and take an anodyne at bed time.

23d. Purging and tenesmus gone. Omit calomel and take a full diet. An anodyne at night.

24th. Recovering strength fast. Dismissed.

Case III. When at Madras, Miss Jeffreys, a delicate girl, aged six years, seemed to be attacked with that modification of the dysentery, which medical writers have called *fluxus celiacus*. The stools were of a perfectly white transparent mucus. Her appetite was squeamish, had feverish symptoms and a very distressing tenesmus The calomel and opium pills with nourishing condiments were given for ten days, and the disease disappeared. Eight other children lived in the same cabin, none of whom had the complaint.

Case IV. As an evidence of the efficacy of mercury in chronic dysentery, I shall relate the following case of Mary Armstrong, aged 20. During her pregnancy at the Cape of Good Hope, she had been attacked with remittent fever and the dysentery, most probably induced by a debilitated system, when under the operation of mercury for lues venerea. She was harrassed by this combination of diseases at the time that the child was born. She did not make any application till a month after she came on board. Her complaints were then, repeated purging of mucus and blood, slight febrile symptoms, and debility. The child, worn out by the same distemper, died when about six months old, in extreme misery. The pills of calomel and opium were immediately prescribed for the mother, and good diet and port wine liberally allowed. The next day the purging and tenesmus very little abated, and she was excessively languid. The medicines continued. From this time to the eighteenth day, March 20, the purging and griping gradually diminished; and as her mouth and throat were slightly affected by the calomel, a pill consisting of extract opii, gr. jß. cerus. acet. gr. j. pulv. zinizber, gr. iij. was ordered to be taken, and repeated twice a day. The medicines were continued till the 24th, when becoming free from every troublesome symptom, she was dismissed, and had no relapse during the remainder of the passage.

Case V. Samuel Rosey, aged 17. When first attacked with the dysentery, on the 11th of June, at Diamond Harbour, bour, he passed about thirty evacuations a day, of a mucous and purulent kind, with great pain and tenesmus. Calomel was administered; but the next day he went to Calcutta, and did not return till the 19th, during which time he had had frequent stools with ascarides and violent gripings and tenesmus. Calomel c. opio.

21st. Appetite bad; countenance sallow; purging and other symptoms as yesterday.

22d. The same, with acute pain in the left hypochondrium, and weak pulse. Sago and wine.

23d. Pains of the side and purging abated.

24th. Has had but two or three evacuations the last twelve hours. Mouth affected. Has very distressing griping pains. Take a draught with tinct. opii et sp. vol. c. c. aa. gtt. L. twice a day, and omit the calomel.

25th. No complaint.

A detail of cases exactly similar would be tedious and uninteresting, otherwise fifty might be given, which yielded to calomel and opium alone. Those treated by other medicines were lingering and obstinate. And though experience proves the superior efficacy of mercury in this disease to a demonstration, cases will occur where it cannot be administered so extensively as could be wished. In those of the chronic form, attended with universal agonizing pains and great emaciation, the almost exclusive use of opium becomes indispensable. Of this description is the following of fatal termination.

Case VI. T. Phillips, aged 22, of the 34th regiment, had, just before he came on board, laboured under fever and the dysentery. He was highly debilitated, and the crowded ship, combined with very bad weather, increased the violence of his malady. From the commencement he had that dejection and expression of countenance, described by Hippocrates, and from the 2d of March to the 19th, he continued painfully wasting away with very transient intervals of ease. The dose of opium had been gradually increased; but as a circumstantial detail would be unimportant, the annexed statement from the 19th to his death may be sufficient.

20th. Has a hoarse cough, and spits much. The eyes sunk; he is blind of the left, and can see but indistinctly with the right eye. Sallow countenance, and has a hectic glow on his cheeks. Teeth and lips covered with an incrustation of black matter. Pulse very feeble. Takes a grain and a half of opium every three hours. Wine and sago ad libitum.

21st. Pulse scarcely perceptible. Purging almost constant. Has taken nothing but a little wine all day.

22d. In the same hopeless state as yesterday. An anodyne at night.

23d. Last night about twelve he was seized with severe pains and griping. Has vomited a quantity of bilious matter, and has recovered in some measure the sight of the left eye. Towards evening he has had universal pains; purging very frequent and bloody. Take two grains of opium every three hours.

24th. Pulse full and rather quick. In very little pain, and drinks plentifully of wine. Has had only three stools to day. Continue the medicine.

25th. Feels a numbness all over him and a deathy sensation about the heart. Purging seldom, and the pulse rather feeble. Expectorates a frothy mucus.

26th and 27th. Much the same as on the preceding day, except the addition of singultus.

28th. Pulse pretty full; spits a viscid mucus. Has a ghastly appearance, and is wasted almost to a skeleton. The liberal use of port wine has had a good effect in keeping the spirits and pulse from sinking. On the 21st, 22d, and 23d, when he could not take the usual quantity of opium and but little wine, the consequences proved the absolute necessity of their administration.

29th. Last night, about ten o'clock, he was attacked with constant purging. He seemed nearly suffocated by the mucus in his throat, and this morning his head and bowels are much affected. In the evening he expired.

I had imagined whenever the disease put on such a dreadful appearance, that no advantage could be possibly derived from medicine; but W. Raffarty, another chronic patient, labouring under symptoms equally formidable at the same time, recovered by exactly the same treatment. With the dysuria he had a complete phimosis,* which disappeared as the original disease wore off. The tormina of the bowels was, if possible, more exquisite than in the case of

* The phimosis occurred when the throat was very much affected. Could this arise from sympathy or associated motions between the fauces and genitals, as in parotitis, hydrophobia, hanging? It is certain that there was no venereal infection whatever, and perhaps any morbid action in the pelvis could not have induced such a symptom.

of Phillips, and the evacuations of blood much more considerable.

Case VII. Joseph Henricks, poulterer, aged 20, was just recovering from fever and the dysentery when he went to Calcutta, and there, from drinking excessively of Bengal rum undiluted, and exposing himself during intoxication to the alternate influence of atmospheric heat and moisture, the disease returned with redoubled violence. From the 30th of July to the 4th of August, he had considerable purging, nausea, tenesmus, and loathing of food. On the 5th he was much reduced. His complexion was sallow and cadaverous; the pupils contracted to a point; his pulse quick; and had excessive thirst. Take four grains of calomel three times a day, and wine and congee for common drink.

6th. Had an anodyne last night with tinct. opii gtt. xl. The pupils more dilated; the purging diminished, but still mucous and purulent. Great pain in the pelvis and bladder, with dysuria. Towards the evening the tenesmus was excruciatingly severe, and nothing voided but a white slimy material. Pulse weak and his spirits sunk. Had an oleaginous aromatic draught, but it was instantly rejected. Ordered forty drops of laudanum to be taken every six hours.

7th. Every way better this morning. Had but two or three stools last night. Has taken some tea and he seems refreshed. Pain of the bowels returned. Apply to the abdomen a volatile liniment with tincture of opium. In the afternoon the evacuations consisted of mucus mixed with blood. Latterly no food or medicine has remained on the stomach. Repeat the draught, and add to each, ol. menthæ ppt. gtt. vj.

8th. Stools are again nothing but mucus. Pupil still contracted, and the pulse small. Debility of the stomach continues. To have a blister over the region of the stomach. Immerse the feet and legs in warm water twice a day, and take two grains of calomel every four hours. Has complete loathing of food, wavering and confusion of head.

9th. Blister rose well. Pulse quick and more full. Pupils more dilated. Is now perfectly collected. Purging the same, but with much less pain. With each dose of calomel take fifteen drops of laudanum.

10th. Pulse very weak. Continual purging in the night, but with less pain than usual. Refuses all kind of food and nourishment. Great despondency. Take tinct. opii gtt. lx. in wine, and if retained, to be repeated every six hours. In the night he had cold and clammy perspiration; feeble pulse; purging nothing less. Add to each draught elixir vitriol gtt. v.

11th. The pulse still small, and the purging greatly diminished. Has a little appetite and is free from pain. Passes very little urine.

12th. Yesterday the purging was trifling, but increased in the night; almost every motion of the body or change of posture producing an inclination to go to stool. This morning he is much emaciated. His countenance is meagre and sallow, pupil contracted, and very weak pulse. Vibices appear on the breast. Stomach still irritable. Had an anodyne aromatic draught, which was vomited up a few minutes after it was taken. Has a ghastly aspect. Pupils more contracted, and the pulse hardly perceptible. Obstinately refuses nourishment. About a pint of coagulated blood was passed at one stool. He is very restless. Take half a drachm of the bark, and five drops of laudanum. All the day he has passed by stool the same bloody material. Has great confusion of intellect. Repeat the bark every two hours.

13th. Pulse very feeble. Pupil dilated more ~~than~~ in health. Frequent stools of clotted blood. In the middle of the day the purging diminished. Spits a good deal and sometimes blood. Absolutely refuses both medicine and food. At nine P. M. he died without the least symptom of pain.

### Appearances on Dissection.

I opened the body ten hours after death. The vesicula fellis was much larger than usual, and distended with bile, which had strongly tinged the peritoneum, stomach and colon. On evacuating the gall bladder, I found the ductus communis choledochus almost impervious. It had in all probability become paralytic from the excessive stimulus of burning spirits he had so imprudently swallowed. There was no appearance of bile where the duct enters the duodenum. The liver was of a brownish leaden colour, very firm, and of the natural size. On making an incision along the small intestines, the glandulous coat was eroded in many parts, and covered with a viscid dark coloured slime. The colon was considerably ulcerated, and studded with livid purple spots. The rectum* was thickened and ulcerated

* See the drawing.

ated more than the colon, and contracted to half its diameter. Cutting into the fat between the bladder and rectum, there appeared intersections of membranous filaments loaded with hydatid-like vesicles, from which rushed out a transparent fluid. The bladder contained about two ounces of muddy urine. The prostate gland was enlarged, and the urethra, towards the neck of the bladder, much contracted. The stomach had nothing in it but a little brownish coloured fluid, and the mucous membrane was much abraded. The whole intestinal tube was empty of every thing but wind. The other viscera had no marks of disease.

You will observe, Sir, that I have here, contrary to general practice, placed the fatal cases in the most conspicuous point of view; and though they furnish little argument against the above mentioned treatment, they illustrate sufficiently the deadly tendency of the disease. As all theory is supported by a general collection of inferences, legitimately drawn from facts, that must be the most correct and comprehensive which can be applied to the greatest number of cases. For however humiliating it may appear to medical men, our brightest prospects will be often overcast, and the most approved means of cure will be occasionally ineffectual. Then we may truly say with the sententious Euripides, *Τι βροτος φρονεω λεγω.* No medicine was ever discovered to be uniformly successful in any complaint, and it would be idle and presumptuous to recommend one possessing these qualities in the dysentery. Those who, with self complacency, publish to the world the invariable success of particular practice, must conscientiously know what numbers are dying in secret, and must certainly feel, in desperate cases, the inutility of all human exertion.

The method of treatment here pointed out, rests upon the solid basis of experience, unaided by the seducing embellishments of hypothetical reasoning. The indulgence of imagination in the construction of theories, has certainly very considerably impeded the progress of practical science; for admitting the ingenuity and originality of those theories, they have only flourished to be forgotten; and the most illustrious speculative physicians on record, have left very little behind them of essential benefit to mankind.

### APPENDIX.

For the particulars of the following dissection I am indebted to Mr. Ashton, an ingenious surgeon in the honora-

 ble ble Company's service, to whom the method of treatment above recommended was suggested, and which he has found peculiarly advantageous. The subject of the dissection was a soldier on his passage from India, aged 35, of an impaired constitution and meagre aspect. He had suffered severely from syphilis, and had a bony tumour on each tibia. He was invalided for what he called "the pains in his bones." He died of the severe dysentery.

*Dissection, Six Hours after Death, February* 14, 1804, *at St. Helena.*

On opening the abdomen by the common crucial incision, the omentum appeared nearly obliterated, and what remained was deprived of its adipose substance.* The jejunum and ileum were in a high state of inflammation,† and looked as if their vessels were distended by a minute injection, but there was no adhesion of parts; their internal surface was very much diseased and gangrenous, not even the size of a sixpence being free from ulceration. The muscular coat was considerably discoloured. The duodenum had no external inflammation, but internally it was much the same as the jejunum and ileum.

The affection of the colon about the sigmoid flexure was similar to the small intestines; the villous coat was soft, and easily separated by a slight touch. Between the rectum and the transverse arch, there were several contractions,‡ some of them so small as only to admit the passage of the little finger. Above them were some black fæces but not hard or scyballous. The rectum being slit up, also exhibited a more

* He complained during his illness of cutting pains under the umbilicus.

† If this was inflammation, to what range or class of diseases must it be referred? Is it to be cured by the antiphlogistic regimen? Inflammations arise from abundance of blood; but is it inconsistent with all medical facts to conjecture, that they may also arise from a penury of blood? This man was emaciated and infirm to the last degree, and still the dissection proves that the intestines were inflamed, "as if their vessels were distended by a minute injection." In this diffused redness there is something essentially different from common inflammation, or such as depends upon sthenic diathesis. Pain arises from diminished as well as increased action; and as I think no physician would employ the remedies usual in inflammations under the circumstances of the above case, and as invigorating the system must be the true indication of cure, there can be little doubt that the two inflammations depend upon very opposite causes. Perhaps it is the peculiarity of asthenic inflammation to terminate in gangrene.

‡ This contraction is a consequence and not a cause of the disease, as Dr. Cullen imagined.

a more diseased appearance, ulceration having nearly penetrated through the coats. The mesentery was highly inflamed, and some of its glands increased to the size of a large almond, hard but not schirrous. The stomach contained about half a pint of a brown frothy fluid, and surrounding the pylorus; it was in a state similar to the duodenum. The liver was of a brown stony colour, soft and rotten, separating between the fingers. The gall bladder* was much distended, and contained at least seven ounces of very black, thick, ropy bile. The pancreas was much smaller and harder than natural. The spleen and kidnies seemingly as in health. There was no preternatural appearance in the thorax; only the heart was very lean, small, and firmly contracted.

*London, May* 1, 1804.

* These appearances of the liver and gall-bladder illustrate an opinion advanced in the former part of this work. No mention is made of the state of the vesica urinariæ. Parts of the diseased viscera were shewn me a few hours after dissection.

## Figures and Tables

**Figure f1:**